# Stereotyping across intersections of race and age: Racial stereotyping among White adults working with children

**DOI:** 10.1371/journal.pone.0201696

**Published:** 2018-09-12

**Authors:** Naomi Priest, Natalie Slopen, Susan Woolford, Jeny Tony Philip, Dianne Singer, Anna Daly Kauffman, Kathryn Mosely, Matthew Davis, Yusuf Ransome, David Williams

**Affiliations:** 1 Centre for Social Research and Methods, College of Arts and Social Sciences, Australian National University, Acton, Australian Capital Territory, Australia; 2 Department of Epidemiology and Biostatistics, University of Maryland, College Park, Maryland, United States of America; 3 Department of General Pediatrics, University of Michigan, Ann Arbor, Michigan, United States of America; 4 Munich Center for the Economics of Aging (MEA), Max-Planck-Institute for Social Law and Social Policy, Munich, Germany; 5 Ann & Robert H. Lurie Children’s Hospital, Chicago, Illinois, United States of America; 6 Yale School of Public Health, Department of Social and Behavioral Science, New Haven, Connecticut, United States of America; 7 Department of Social and Behavioral Sciences, Harvard T H Chan School of Public Health, Harvard University, Boston, Massachusetts, United States of America; 8 Department of African and African American Studies and of Sociology, Harvard University, Cambridge, Massachusetts, United States of America; 9 Department of Psychiatry and Mental Health, University of Cape Town, Cape Town, South Africa; York University, CANADA

## Abstract

This study examined the prevalence of racial/ethnic stereotypes among White adults who work or volunteer with children, and whether stereotyping of racial/ethnic groups varied towards different age groups. Participants were 1022 White adults who volunteer and/or work with children in the United States who completed a cross-sectional, online survey. Results indicate high proportions of adults who work or volunteer with children endorsed negative stereotypes towards Blacks and other ethnic minorities. Respondents were most likely to endorse negative stereotypes towards Blacks, and least likely towards Asians (relative to Whites). Moreover, endorsement of negative stereotypes by race was moderated by target age. Stereotypes were often lower towards young children but higher towards teens.

## Introduction

Children from racially stigmatised and ethnic minority groups experience substantial inequalities across a range of health and development indicators globally, with these patterns of unequal burden of disease continuing into adulthood [1–4]. Understanding how and why these racial/ethnic inequalities occur and persist is now widely considered incomplete without attention to possibly the most critical and distinctive social exposure experienced by stigmatized racial/ethnic groups—the added burden of racism [5–8]. Racism is an organized system of oppression built on the social categorization and stratification of social groups into ‘races’ that devalues and disempowers those groups regarded as inferior and differentially allocates to them valued societal opportunities and resources [9, 10]. Operating across intrapersonal (internalised), interpersonal, and systemic/structural domains, racism can be expressed in multiple forms [11] including as stereotypes (categorical beliefs), prejudice (negative attitudes), and discrimination (unequal treatment) [12]. Attention to racism as a fundamental cause of health inequalities [7, 13] is even more pertinent for children and young people who are particularly vulnerable to racism’s harmful effects [14, 15].

Growing evidence documents negative effects of racism during pre-conception, pregnancy and birth, early and middle childhood, through to adolescence [8, 15, 16]. Among children and youth racism has been associated with a range of negative mental health outcomes [17, 18], indicators of poor physical health including allostatic load [19], immune, inflammatory and chronic disease biomarkers [20–22], as well as social and cognitive development [23]. This evidence is consistent with wider scientific consensus that early life experiences and exposures play a substantive role in later outcomes and inequalities [24].

Racism can influence child health and development through multiple pathways. Institutional and cultural racism can harm health through stigma, stereotypes, prejudice and racial discrimination, all of which can lead to differential access to a broad range of societal resources and opportunities required for health [25]. Perceived or self-reported discrimination–defined as a behavioural manifestation of a negative attitude, judgement, or unfair treatment towards members of a group–is also an important yet often neglected psychosocial stressor with substantial deleterious health impacts throughout life [26]. Children and young people may experience racism both directly, where they themselves are the targets of racism, or vicariously, where their parents, caregivers, family and peers experience racism that children and youth may or may not witness or perceive [18, 27]. With the evidence base still emergent, investigating patterns and impacts of exposure to racism early in life, and causal mechanisms and processes by which such exposures influence later outcomes, remains critically important [15, 25]. However, an equally critical task is to understand, prevent, and reduce sources and expressions of racism in the daily lives of children and youth. This includes understanding the racial/ethnic beliefs and attitudes of non-parent/primary caregiver adults who provide care and services to children and young people.

Racial/ethnic beliefs and attitudes are strongly predictive of conscious and unconscious behaviors, including levels of active helping and passive neglecting [28] and verbal and non-verbal behaviors such as word choice, verbal tone, eye-contact, degree of interpersonal distance and facial expressions [29]. When present among those who provide care and services to children and youth, their families and communities, such racial bias has been shown to result in differential treatment and poorer outcomes for those from stigmatized racial/ethnic groups across a range of settings including health and healthcare [30], teaching methods and classroom practices [31], academic expectations and judgement of ability and attainment [32], school discipline [33], housing and neighborhood quality [34], employment and workplace conditions [35], policing [36] and criminal justice processes [37]. However, patterns of racial beliefs and attitudes of adults towards different age groups, including children and youth, is relatively under-explored despite being critically important to the lives of those from stigmatized racial/ethnic backgrounds.

Considerable work now exists in the United States (U.S.) that documents racial beliefs and attitudes of White Americans towards those from stigmatised racial ethnic backgrounds over time [38, 39]. However, few such studies of stereotyping of racial/ethnic groups have investigated how these perceptions may vary towards different age groups. One study found elderly African Americans were judged less harshly than younger African Americans, with negative racial stereotypes of hostility and danger lessened by age-related stereotypes of frailty and kindness [40]. Another has found that compared to African American adolescents, African American children were perceived with fewer negative racial stereotypes, and subsequently less likely to be penalized [41]. These studies suggest that stereotypes related to both old-age and to childhood are somewhat protective against negative racial stereotypes towards African Americans, though in themselves stereotypes regarding old age [42] and childhood [43] can still be highly problematic. Even appearing to be young, in contrast to actually being young, has been shown to act as a form of threat diffusion, cueing warmth and attenuating stereotypical perceptions of Blacks as threatening [44]. Findings from one study demonstrated Black Chief Executive Officers (CEO)s with childlike facial features, that is a baby face, were perceived as warmer than mature-faced Black CEOs, although overall ordinary Blacks were rated as less warm than ordinary Whites. However, others have found in experimental studies that characteristics often associated with childhood such as innocence and need for protection are afforded to Black children less than they are to White children, and that Black children are still viewed in more dehumanizing ways than White children [45], despite Black children being perceived less negatively and with less racial bias than Black adolescents and adults. This has been shown for Black boys ten years and older [45] and also for Black girls beginning at age five years [46] in relation to their White same-gender peers.

In order to allow for consideration of concurrent interactions between different dimensions of social identity and of the impact of interconnected processes and systems of classification (e.g. racism, ageism) that operate across levels, intersectional approaches that extend beyond analysis of single social categories (i.e. race, age) are thus recommended [47–50].

Intersectional approaches recognise single social identities and categories are not independent but instead intersect and interact impacting both social perceivers’ impressions, and social targets’ experiences, and are increasingly being applied within the quantitative health and social sciences [51, 52]. Such approaches are highly relevant to studying stereotypes, which frequently include components that are both positive and negative [28]. As identified in the stereotype content model (SCM), stereotypes of specific social groups, including racial/ethnic groups, are focused across the two dimensions of warmth (e.g. warm, sincere) and competence (e.g. capable, competent) with different target groups defined across low versus high warmth and competence. For racial/ethnic minorities, African Americans and other stigmatised groups such as Latinos, Arab Americans, and indigenous peoples, stereotypes tend to be both low in perceived warmth and competence while Asians tend to be stereotyped as cold yet competent. How then do these racial/ethnic stereotypes intersect with age-related stereotypes? Children tend to be stereotyped as high in warmth but low in competence, while teenagers are more likely to be seen as low in both warmth and competence. Do age-related stereotypes diffuse or exacerbate stereotypes towards racial/ethnic groups? How does this differ for different racial ethnic groups?

This study aimed to investigate 1) prevalence of racial/ethnic stereotypes among White adults who work or volunteer with children; and 2) whether stereotyping of racial/ethnic groups is moderated by target age. Drawing on previous research, we hypothesised that, Blacks, Latinos, Arab Americans, and indigenous peoples would all be more likely than Whites to be perceived as lazy, violence prone, unintelligent, and having unhealthy habits, while Asian Americans would be perceived less likely to have these characteristics. Informed by studies showing age-related stereotypes can act as a form of threat diffusion, cueing warmth and attenuating stereotypical perceptions of stigmatized ethnic groups, we hypothesised that target age would moderate the association between negative stereotypes and race/ethnicity. Specifically, that negative stereotype endorsement towards Blacks, Latinos, Arab Americans and indigenous peoples would be lower for young children than for adults, and lower for teens than for adults, though to a lesser extent than for young children.

## Materials and methods

### Participants

Participants were 1022 White civilian, non-institutionalized adults (64% female, 36% male) in the U.S (ages 18–83, M = 51 years, SD = 15 years). All participants worked and/or volunteered with children (52% only volunteered, and 48% worked and volunteered or only worked, with children).

### Procedure

The National Voices Project (NVP), sponsored by and conducted in partnership with the W.K. Kellogg Foundation, was a recurring internet-based nationally representative sample of adults who work and/or volunteer with children. It was conducted to identify concerns about children’s health, education, and economic security at the community level. The NVP 3 survey utilized in this present study was fielded August 27- September 30, 2013, by a survey contractor, GfK Custom Research, LLC Group (GfK) to its web-enabled KnowledgePanel^®^ and KnowledgePanel Latino® probability-based panels. Panel participants were initially invited through random selection of telephone numbers and residential addresses; panel members were invited to participate in the present study through random selection. In addition, two opt-in e-Rewards panels run through GfK were also used to supplement KnowledgePanel® in this study, in order to oversample for adults working in racially/ethnically diverse urban centers previously identified by the W.K. Kellogg Foundation for another initiative called “Place Matters” that was not connected to the National Voices Project or to the study questions posed here. Using an iterative SAS raking procedure, controlling for demographics within White and Non-White, weights were computed for the combined KnowledgePanel® on-panel cases and the opt-in off-panel qualified respondents [53]. As a result of this weighting task the blended samples (on-panel and opt-in off-panel) approximate the benchmarks of the KnowledgePanel® on-panel only sample [53]. Supplementary analysis provided online shows minimal difference in estimations using on-panel and off-panel sample using sample weights.

Respondents in sampled households were invited to complete a brief screening questionnaire to determine their occupational and/or volunteer eligibility. The survey was offered in both English and Spanish. This method identified an eligible sample of 2,613 adults from 50 states and the District of Columbia who were at least 18 years old, who represented 15% of those initially contacted to participate. Of the overall eligible sample, 1022 self-identified as non-Hispanic White (606 on-panel and 416 off-panel respondents). This was the analytic sample for the current study. The University of Michigan Institutional Review Board declared this study exempt from human subjects review. The IRB study number is: National Voices Project (HUM00049778). Informed consent was obtained from participants recruited to the online Knowledge Panel to receive ongoing survey invitations. Knowledge Panel participants were then invited to participate in the National Voices Project survey via email that included a link to the survey information.

### Measures

#### Demographic information

In addition to race/ethnicity, all participants reported their own gender (male, female), age, and education (less than high school, high school, some college, bachelor’s degree or higher).

#### Racial/Ethnic stereotypes

Stereotype endorsement was assessed using items adapted from the General Social Survey (GSS) [39, 54]. Participants were asked to rate characteristics of different groups of people on a scale of 1 to 7 with instructions *‘A score of 1 OR 7 means that you think almost all of the people in that group have that characteristic*. *A score of 4 means you think that the group is not towards one end or another*.*’* Characteristics rated by participants related to common stereotypes: lazy/hardworking; violent/not violence prone; unintelligent/intelligent; unhealthy habits/healthy habits. (Note: unhealthy habits stereotype was added for this study and was not included in the original GSS.) All participants were asked questions about Whites, African Americans/Blacks (Blacks), and Hispanic/Latinos (Hispanic); respondents were randomized to be asked about one of four additional race/ethnicity categories: American Indian/Alaska Natives (AI/AN); Asian/Asian Americans (Asian); Pacific Islanders/Native Hawaiians (PI/NH); Arab/Arab Americans (Arab). (Note: Abbreviations in parentheses are used hereafter for brevity.)

#### Target age group

All participants were asked to rate adults in relation to the four stereotype characteristics. Participants were randomized to also rate either ‘young children age 0–8’ or ‘teens age 13–18’ in relation to these stereotypes. E.g. survey questions read: *‘Please rate whether young children age 0–8 in each group tend to be hard-working or lazy*.*’; Please rate whether teens age 13–18 in each group tend to be hard-working or lazy*.

### Analysis

First the proportion of respondents endorsing each negative stereotype for each target age group was calculated. The seven response options were collapsed into a trichotomous analytic variable: yes v neither v counter (neither represented a score of 4) following methods previously reported with these stereotype measures [55, 56] and to capture the ‘neither’ response following methods previously reported using racial attitudes in the U.S. that highlight the importance of this response category [39].

For the multivariable analysis, we created a binary variable for each negative stereotype: comparing those who responded ‘yes’ with those responding ‘neither/counter’ collapsed in one group. The ‘neither’ ratings were grouped with the ‘counter’ responses to allow focus on those responding ‘yes’ to the negative stereotype following previous approaches [39]. Sensitivity analysis was conducted using multinomial regression to examine the three-category stereotype variables (yes, neither, counter) and assess appropriateness of the two-category stereotype variables (yes, neither/counter). As findings were consistent across the three-category and two-category analyses, the two-category variable was used for a more parsimonious approach with more straightforward interpretation. Supplementary models (provided online) were run using the original seven-point measure for both the prevalence and multivariable analysis to reduce risk of bias or loss of data resulting from re-coding of such variables. As patterns of association remained consistent with analysis using the dichotomized variables, the dichotomized variables were used for a more interpretable approach than the seven-point measure and for consistency with previous studies as noted above [39, 55, 56].

Prevalence rate ratios (PRR) for each stereotype were calculated using generalized estimating equations using the poisson distribution and log link. PRRs were adjusted for respondent age, gender and education level. To test our hypothesis that target age moderates the association between stereotype rating and race, we analyzed effect modification using both additive and multiplicative scales [57–59], estimates of the PRR within strata of target age with a single reference category of adult/White were calculated, as well as the PRR within strata of target age. As recommended, measures of effect measure modification (EMM) on both the additive and multiplicative scale were then computed [57]. Evidence of a multiplicative EMM was examined through a cross-product term (ageXrace) in the models. This equals 1 in the absence of multiplicative EMM. Presence of an additive EMM was assessed using the relative excess risk of interaction (RERI). RERI represents the prevalence risk that is in excess of that expected if the combination of target age and race were entirely additive [57]. In the absence of additive EMM, RERI is equal to 0 [58, 59].

All analyses were conducted in Stata/MP 14 using the ‘svy’ commands to accommodate sample design characteristics including sampling weights and panel design. GfK provided the sampling weights based on probabilities of initial invitation to participate in the study and of response.

## Results

### Proportion of White adults who work and/or volunteer with children endorsing racial/ethnic stereotypes

As shown in Table 1, White adults who work or volunteer with children had higher levels of endorsement of negative stereotypes for Black adults than for adults in any other racial/ethnic group—violence prone (52%), unintelligent (19%), and to have unhealthy habits (36%). Only AI/AN were endorsed more frequently by Whites regarding being lazy (30%; versus 24% perceiving Blacks as lazy). Far fewer White respondents endorsed negative stereotypes towards White adults than towards all other racial/ethnic groups, except toward Asian adults; PI/NH adults were considered less violence prone (5%) and to have fewer unhealthy habits (15%), as were Arab adults (10%) compared to White adults. Black teens were viewed just as negatively as Black adults by respondents, with AI/AN teens and Hispanic teens seen at similarly negative levels on several stereotypes. Respondents were more likely to view Black teens as lazy than then Black adults (41% v 24%). Of note, very low levels of endorsement of the “not violence prone” stereotype for Blacks and Hispanics were also observed (see Table 2). Compared to White adults’ views of Black adults, young Black children (see Table 3) were viewed less negatively: lazy (14%), violence prone (23%), unintelligent (11%) and to have unhealthy habits (38%) versus consistently higher proportions for Black adults. Young Black children were, however, perceived more negatively than young children from other racial/ethnic groups except for AI/AN and Hispanic young children.

**Table 1:** Population weighted estimates of the proportion of White adults who work or volunteer with children endorsing stereotypes towards adults, by racial group.

	White % (95% CI)	Afr. Am. % (95% CI)	Hispanic % (95% CI)	AI/AN % (95% CI)	Asian Am. % (95% CI)	PI/NH % (95% CI)	Arab Am. % (95% CI)
	n = 1004	n = 1005	n = 1004	n = 244	n = 249	n = 265	n = 241
**Hardworking or Lazy**							
Hardworking	56.92 (51.01, 62.63)	35.69 (30.3, 41.47)	59 (53.14, 64.61)	22.28 (14.67, 32.35)	72.66 (61.03, 81.84)	45.41 (34.8, 56.45)	38.35 (27.4, 50.62)
Neither	38.79 (33.21, 44.67)	40.32 (34.72, 46.18)	31.08 (25.94, 36.72)	47.1 (35.91, 58.6)	24.65 (15.94, 36.06)	48.03 (37.11, 59.14)	48.68 (36.88, 60.63)
Lazy	4.3 (2.46, 7.41)	23.99 (19.31, 29.4)	9.93 (6.95, 13.99)	30.61 (20.53, 42.96)	2.7 (0.68, 10.16)	6.57 (2.7, 15.11)	12.97 (6.45, 24.36)
**Not violence prone or violence prone**							
Not violence prone	20.79 (16.47, 25.88)	10.09 (7, 14.33)	12.06 (8.78, 16.36)	16.27 (9.34, 26.83)	45.95 (34.03, 58.34)	25.85 (17.51, 36.4)	25.28 (15.82, 37.84)
Neither	57.33 (51.43, 63.03)	38.09 (32.61, 43.9)	44.53 (38.83, 50.39)	54.9 (43.13, 66.14)	44.51 (33.02, 56.63)	68.94 (58.14, 78.01)	46.2 (34.62, 58.21)
Violence prone	21.88 (17.32, 27.26)	51.82 (45.96, 57.63)	43.4 (37.67, 49.32)	28.83 (18.96, 41.23)	9.54 (3.79, 22)	5.21 (2.19, 11.92)	28.52 (18.8, 40.74)
**Intelligent or Unintelligent**							
Intelligent	41.78 (36.16, 47.61)	28.18 (23.39, 33.52)	23.25 (18.85, 28.33)	22.72 (14.96, 32.96)	53.07 (40.89, 64.9)	33.96 (24.47, 44.96)	33.59 (23.37, 45.61)
Neither	48.48 (42.63, 54.37)	52.65 (46.8, 58.43)	54.81 (48.94, 60.54)	60.24 (48.45, 70.95)	39.04 (28.01, 51.32)	56.31 (45.29, 66.74)	52.37 (40.32, 64.16)
Unintelligent	9.75 (6.69, 14)	19.17 (14.94, 24.25)	21.94 (17.41, 27.27)	17.04 (9.43, 28.83)	7.88 (3.06, 18.81)	9.72 (5.3, 17.18)	14.04 (7.09, 25.91)
**Healthy or Unhealthy habits**							
Healthy habits	29.13 (24.15, 34.67)	14.56 (10.85, 19.25)	17.1 (13.08, 22.04)	13.99 (7.93, 23.5)	46.21 (34.31, 58.57)	25.45 (17.29, 35.79)	25.97 (16.59, 38.23)
Neither	46.62 (40.84, 52.49)	49.19 (43.37, 55.04)	51.09 (45.22, 56.93)	50.41 (39.05, 61.72)	40.63 (29.58, 52.71)	59.47 (48.51, 69.56)	63.71 (51.12, 74.67)
Unhealthy habits	24.25 (19.45, 29.79)	36.25 (30.83, 42.04)	31.81 (26.53, 37.6)	35.6 (25.19, 47.58)	13.16 (6.72, 24.18)	15.08 (9.13, 23.91)	10.32 (4.6, 21.53)

**Table 2:** Population weighted estimates of the proportion of White adults who work or volunteer with children endorsing stereotypes towards teens, by racial group.

	White % (95% CI)	Afr. Am. % (95% CI)	Hispanic % (95% CI)	AI/AN % (95% CI)	Asian Am. % (95% CI)	PI/NH % (95% CI)	Arab Am. % (95% CI)
	n = 491	n = 494	n = 493	n = 123	n = 127	n = 129	n = 133
**Hardworking or Lazy**							
Hardworking	28.96 (22.11,36.92)	19.98 (14.11,27.52)	27.97 (21.08,36.07)	18.03 (9.28,32.13)	54.51 (36.89,71.07)	28.87 (17.17,44.27)	20.18 (10.35,35.62)
Neither	40.1 (32.46,48.26)	38.61 (31.02,46.79)	45.33 (37.39,53.51)	37.61 (24.09,53.37)	38.52 (23.1,56.64)	55.65 (40.11,70.16)	57.69 (41.36,72.5)
Lazy	30.94 (23.71,39.24)	41.41 (33.57,49.72)	26.71 (20.05,34.62)	44.36 (29.06,60.81)	6.97 (2.24,19.71)	15.48 (6.79,31.54)	22.13 (11.04,39.44)
**Not violence prone or violence prone**							
Not violence prone	20.14 (14.53,27.22)	9.02 (5.4,14.69)	8.96 (5.53,14.18)	14.41 (6.55,28.77)	33.18 (18.99,51.27)	20.72 (11.5,34.46)	10.02 (3.7,24.37)
Neither	55.54 (47.29,63.49)	40.77 (32.97,49.06)	48.93 (40.86,57.06)	57.42 (41.17,72.21)	59.99 (42.16,75.51)	68.37 (53.34,80.35)	66.66 (49.95,80.02)
Violence prone	24.32 (17.86,32.21)	50.21 (42.1,58.3)	42.11 (34.32,50.32)	28.17 (15.52,45.58)	6.83 (2.11,19.96)	10.9 (4.43,24.4)	23.33 (12.22,39.95)
**Intelligent or Unintelligent**							
Intelligent	42.21 (34.36,50.48)	28.85 (21.86,37.02)	28.37 (21.38,36.59)	35.84 (21.85,52.73)	56.17 (38.58,72.33)	31.77 (19.5,47.24)	29.85 (16.77,47.33)
Neither	52.04 (43.87,60.1)	52.14 (43.95,60.22)	52.22 (44.06,60.27)	55.93 (40.04,70.69)	30.33 (17.71,46.83)	58.2 (42.89,72.08)	62.71 (45.65,77.1)
Unintelligent	5.75 (3.29,9.87)	19.01 (13.46,26.15)	19.4 (13.84,26.51)	8.23 (3.64,17.59)	13.5 (4.62,33.47)	10.02 (4.63,20.38)	7.44 (2.32,21.39)
**Healthy or Unhealthy habits**							
Healthy habits	31.9 (24.64,40.16)	23.74 (17.11,31.96)	23.11 (16.55,31.29)	22.39 (11.14,39.9)	47.06 (29.83,65.01)	29.07 (17.03,44.99)	26.85 (14.36,44.55)
Neither	38.38 (30.86,46.49)	42.81 (34.99,51.01)	46.04 (38.02,54.27)	48.97 (33.51,64.63)	41.15 (25.71,58.56)	50.48 (35.34,65.53)	50.85 (34.95,66.58)
Unhealthy habits	29.73 (22.61,37.98)	33.45 (26.28,41.47)	30.85 (23.79,38.95)	28.64 (16.72,44.52)	11.79 (4.1,29.5)	20.46 (10.92,35.05)	22.3 (11.14,39.67)

**Table 3:** Population weighted estimates of the proportion of White adults who work or volunteer with children endorsing stereotypes towards young children, by racial group.

	White % (95% CI)	Afr. Am. % (95% CI)	Hispanic % (95% CI)	AI/AN % (95% CI)	Asian Am. % (95% CI)	PI/NH % (95% CI)	Arab Am. % (95% CI)
	n = 491	n = 494	n = 493	n = 123	n = 127	n = 129	n = 133
**Hardworking or Lazy**							
Hardworking	26.12 (19.54, 33.99)	18.52 (12.89, 25.88)	22.74 (16.57, 30.38)	17 (7.95, 32.68)	37.52 (23.37, 54.18)	24.72 (13.63, 40.58)	15.85 (6.99, 32.06)
Neither	61.25 (52.78, 69.09)	67.47 (59.1, 74.86)	65.95 (57.65, 73.37)	68.35 (51.7, 81.33)	52.92 (36.33, 68.89)	68.99 (52.84, 81.54)	83.19 (67.17, 92.29)
Lazy	12.63 (7.8, 19.81)	14.01 (8.98, 21.19)	11.31 (6.94, 17.88)	14.65 (6.5, 29.76)	9.55 (2.63, 29.21)	6.29 (1.76, 20.14)	0.96 (0.16, 5.43)
**Not violence prone or violence prone**							
Not violence prone	21.59 (15.81, 28.75)	13.56 (9.03, 19.85)	16.47 (11.31, 23.37)	21.07 (10.69, 37.32)	39.03 (23.92, 56.6)	15.91 (7.64, 30.21)	11.18 (4.18, 26.64)
Neither	69.31 (61.15, 76.42)	63.2 (54.75, 70.91)	67.12 (58.75, 74.53)	64.28 (47.11, 78.43)	58.31 (40.95, 73.83)	78.95 (63.14, 89.15)	81.08 (64.93, 90.85)
Violence prone	9.1 (4.95, 16.14)	23.25 (16.71, 31.38)	16.4 (10.87, 23.99)	14.65 (5.87, 32.08)	2.66 (0.58, 11.4)	5.14 (0.98, 22.82)	7.74 (2.59, 20.91)
**Intelligent or Unintelligent**							
Intelligent	32.87 (25.6, 41.05)	23.01 (16.87, 30.55)	20.57 (14.83, 27.81)	18.77 (9.99, 32.48)	47.9 (31.6, 64.66)	28.53 (16.45, 44.73)	24.61 (12.27, 43.24)
Neither	62.42 (54.12, 70.04)	65.96 (57.74, 73.32)	69.93 (62, 76.83)	71.34 (55.21, 83.4)	46.95 (30.69, 63.89)	68.19 (52.17, 80.81)	71.21 (52.74, 84.58)
Unintelligent	4.72 (2.37, 9.18)	11.03 (6.8, 17.4)	9.5 (5.75, 15.28)	9.89 (3.19, 26.77)	5.15 (1.69, 14.62)	3.29 (1.03, 10.01)	4.18 (0.85, 18.07)
**Healthy or Unhealthy habits**							
Healthy habits	21.91 (15.77, 29.62)	9.57 (5.79, 15.43)	9.9 (6.03, 15.82)	7.71 (2.46, 21.69)	30.65 (17.62, 47.74)	14.01 (6.37, 28.08)	23.05 (11.53, 40.78)
Neither	49.02 (40.68, 57.41)	52.71 (44.27, 61)	58.87 (50.38, 66.85)	55.53 (39.03, 70.9)	58.92 (41.73, 74.18)	61.31 (45.03, 75.41)	53.13 (35.54, 69.98)
Unhealthy habits	29.07 (21.95, 37.38)	37.72 (29.87, 46.26)	31.24 (23.96, 39.58)	36.76 (22.56, 53.7)	10.43 (3.18, 29.21)	24.67 (13.27, 41.21)	23.81 (11.75, 42.32)

### Effect modification of racial/ethnic stereotypes and target race/ethnicity, by age

Table 4 shows the results of the adjusted analyses of EMM. In interpreting these tables, the first set of columns (e.g. ‘Black PRR’ and following) have a reference group of White/Adult, and the final columns (e.g. ‘PRR for being Afr. Am within strata of target age’ and following) are the stratum-specific estimates. For example, in the first set of columns the PRR of 10.16 for Black teens indicates that the risk of Black teens being considered lazy was 10.16 times higher than for White Adults. The PRR for Black teens in strata of target age of 1.32 means that the risk of Black teens being considered lazy was 1.32 times higher than for White teens.

**Table 4:** Modification of negative stereotype endorsement towards racial/ethnic groups by age (Population Rate Ratios (PRR) within strata of target age group with a single reference group of adult/White, and PRR within strata of target age group with White of each target age group as reference group).

	Whites PRR	Black PRR	Hispanic PRR	AI/AN PRR	Asian PRR	Arab PRR	NH/PI PRR	PRR for Black in strata of target age	PRR for Hispanic in strata of target age	PRR for AI/AN in strata of target age	PRR for Asian in strata of target age	PRR for Arab in strata of target age	PRR for NH/PI in strata of target age
**Lazy/Hardworking**													
Adult	1.00	5.58**	2.30**	6.79**	0.61	1.68	3.06**	5.58**	2.31**	6.79**	0.61	1.68	3.06**
Teen	7.71**	10.16**	6.84**	9.79**	1.90	4.42**	5.66**	1.32**	0.87	1.27	0.26**	0.58*	0.73
Child	2.67**	2.95**	2.38*	3.35**	2.01	1.30	0.15	1.11	0.90	1.25	0.75	0.49	0.06*
AdultXTeen EMM mult.		0.24**	0.39**	0.19**		0.35*	0.24**						
EMM add.		-2.14*	-2.21*		-5.21**	-3.30*	-3.81*						
AdultXChild EMM mult.		0.20**	0.39**	0.18**			0.02**						
EMM add.		-4.25**					-5.40**						
**Violent /Non-violent**													
Adult	1.00	2.36**	1.98**	1.20	0.44	0.31**	1.23	2.37**	1.98**	1.20**	0.44	0.31	1.24**
Teen	1.06	2.24**	1.88**	1.34	0.26	0.55	0.93	2.10**	1.76**	1.25	0.24*	0.51	0.87
Child	0.44**	1.10	0.79	0.54	0.12**	0.37*	0.43*	2.48**	1.77**	1.22	0.28	0.83	0.96
AdultXTeen EMM mult.													
EMM add.													
AdultXChild EMM mult.													
EMM add.		-0.70**	-0.63**			0.56**							
**Unintelligent/Intelligent**													
Adult	1.00	1.94**	2.23**	1.82**	0.80	0.96	1.38	1.94**	2.23**	1.82**	0.80	0.96	1.38
Teen	0.57	1.95**	1.96**	0.94	1.28	1.10	0.64	3.46**	3.47**	1.66	2.27	1.95*	1.13
Child	0.49*	1.13	0.97	1.09	0.61	0.34	0.48	2.30**	1.99**	2.24*	1.18	0.70	0.98
AdultXTeen EMM mult.					2.85**								
EMM add.					0.93**								
AdultXChild EMM mult.													
EMM add.			-0.74**										
**Unhealthy/Healthy**													
Adult	1.00	1.50**	1.32**	1.40*	0.60*	0.65*	0.40**	1.50**	1.32**	1.40*	0.60*	0.65*	0.41**
Teen	1.29*	1.46**	1.34*	1.22	0.60	0.99	0.88	1.13	1.04	0.95	0.47*	0.76	0.68*
Child	1.11	1.45**	1.20	1.31	0.44*	0.91	0.96	1.31**	1.08	1.18	0.39*	0.83	0.86
AdultXTeen EMM mult.		0.76**	0.26*										
EMM add.		0.71*											
AdultXChild EMM mult.													
EMM add.		-0.33*											

PRR adjusted for respondent age, gender, education

*p≤0.05.

**p≤0.01

EMM = effect measure modification; estimates for EMM by age on the multiplicative or additive scales only shown for values significant at p < .05.

Note: all comparisons shown in the left panel of columns (e.g. ‘PRR’ columns) have a reference group of White/Adult; the right panel of columns are age stratum-specific estimates, with White adult, White teen, and White child serving as the reference, accordingly.

#### Lazy/Hardworking

All teens, regardless of race/ethnicity, were at higher risk of being considered lazy than were adults. Black teens were at the highest risk (PRR 10.16, 95% CI 5.82, 17.74) compared to White adults, closely followed by AI/AN teens (PRR 9.79, 95% CI 5.11, 18.76). Compared to White teens, Black teens (PRR 1.32, 95% CI 1.09, 1.58) and AI/AN teens (PRR 1.27, 95% CI 0.95, 1.71 were at highest risk of being considered lazy, while Asian teens (PRR 0.26, 95% CI 0.11, 0.58) and Arab teens (PRR 0.57, 95% CI 0.33, 0.99) were at reduced risk. There was strong evidence of race by teen interactions on both the multiplicative and additive scale for all racial/ethnic groups, except for AI/AN where there was weak evidence of interaction on the additive scale, and for Asian where there was no evidence of interaction on the multiplicative scale.

Compared to White adults, young children who were Black (PRR 2.95, 95% CI 1.40, 6.24), Hispanic (PRR 2.38, 95% CI 1.10, 5.16) or AI/AN (PRR 3.35, 95% CI 1.44, 7.75) were at higher risk of being considered lazy. Among young children, NH/PI young children (PRR 0.06, 95% CI 0.00, 1.00) were at reduced risk of being considered lazy compared to White young children. There was strong evidence of race by child interactions on both the multiplicative and additive scale for Black and NH/PI, and strong evidence on the multiplicative scale with moderate to weak evidence on the additive scale for Hispanic and AI/AN groups.

#### Violence-prone/Non-violence-prone

Black (PRR 2.24, 95% CI 1.74, 2.89) and Hispanic (PRR 1.88, 95% CI 1.44, 2.43) teens were at increased risk of being considered violence-prone by respondents compared to White adults, with moderate evidence that Asian teens were at reduced risk (PRR 0.26, 95% CI 0.06, 1.09) compared to White adults. Within teens, Black (PRR 2.10, 95% CI 1.61, 2.75) and Hispanic (PRR 1.76, 95% CI 1.39, 2.23) were at increased risk and Asian teens were at reduced risk (PRR 0.24, 95% CI 0.06, 0.95) of being considered violence-prone compared to White adults. There was no evidence of race by teen interaction on either the multiplicative or the additive scale. Young children who are White (PRR 0.44, 95% CI 0.26, 0.77), Asian (PRR 0.12, 95% CI 0.02, 0.66) Arab (PRR 0.37, 95% CI 0.14, 0.96) and NH/PI (PRR 0.43, 95% CI 0.18, 0.10) were all at reduced risk of being considered violence-prone compared to White adults. Among young children, Black (PRR 2.48, 95% CI 1.61, 3.83) and Hispanic (PRR 1.77, 95% CI 1.16, 2.73) were at increased risk of being considered violence-prone compared to White young children. There was evidence of race by child interactions on the additive scale for Black, Hispanic and Arab but not on the multiplicative scale.

#### Unintelligent/Intelligent

Compared to White adults, Black teens (PRR 1.95, 95% CI 1.24, 3.07) and Hispanic teens (PRR 1.96, 95% CI 1.25, 3.08) were at increased risk of being considered unintelligent. Among teens, Black (PRR 3.46, 95% CI 1.88, 6.36) and Hispanic teens (PRR 3.47, 95% CI 1.90, 6.35) and Arab teens (PRR 1.95, 95% CI 1.05, 3.64) were at increased risk of being considered unintelligent. There was no evidence of race by teen interaction on either scale, except for Asians where there was evidence of interaction on both scales. Among young children, only White young children (PRR 0.49, 95% CI 0.26, 0.90) were at reduced risk of being considered unintelligent compared with White adults. Within young children, Black (PRR 2.30, 95% CI 1.34, 3.94), Hispanic (PRR 1.99, 95% CI 1.23, 3.25), and AI/AN (2.24, 95% CI 1.02, 4.90) were all at increased risk of being considered unintelligent compared with White children. There was no evidence of race by child interaction on either scale.

#### Unhealthy/Healthy habits

In comparison to White adults, White (PRR 1.29, 95% CI 0.99, 1.66), Black (PRR 1.46, 95% 1.26, 1.77), and Hispanic (PRR 1.34, 95% CI 0.99, 1.80) teens were at increased risk of being considered to have unhealthy habits. Within strata of teens, only Asian teens were at reduced risk of being considered as having unhealthy habits (PRR 0.46, 0.22, 0.95). There was evidence of race by teen interaction on both scales for Blacks. For young children in relation to White adults, Black (PRR 1.45, 95% CI 1.12, 1.90) were at increased risk and Asian young children at marginally reduced risk (PRR 0.44, 95% CI 0.19, 1.02) of being considered to have unhealthy habits. Among young children, compared to White children Black children (PRR 1.31, 1.09, 1.58) were at increased risk and Asian children (PRR 0.39, 95% CI 0.17, 0.89) were at reduced risk of being considered to have unhealthy habits. There was statistical evidence of race by child interaction on the additive scale for Blacks.

## Discussion

Racism is increasingly recognized as a fundamental cause of racial/ethnic inequalities in health and development throughout the lifespan. Racial beliefs and attitudes of adults, including those who work or volunteer with children, towards different age groups are likely to have meaningful influence on health inequalities. This study aimed to investigate the prevalence of racial/ethnic stereotypes among White adults who work or volunteer with children in the United States; and whether stereotyping of racial/ethnic groups varied towards adults, teens and young children. Results indicate high proportions of adults who work or volunteer endorsed negative stereotypes towards Blacks and other ethnic minorities. Findings also showed respondents were most likely to endorse negative stereotypes towards Blacks, and least likely towards Asians, and that the proportion of respondents endorsing negative stereotypes were often lower, towards children and were often higher towards teens. This suggests that initiatives to prevent or reduce racial/ethnic inequalities in child health and development should address racial beliefs and attitudes among key adults in children’s lives.

Findings of this study show that White adults who work and/or volunteer with children hold negative stereotypes towards non-White racial/ethnic groups. Moreover, stereotypes persist towards young children and teenagers of minority groups, not only towards adults. Stereotypes were observed at considerable levels towards Black, American Indian/Alaska Native, and Hispanic children and teens among respondents in this study. Positive age-related stereotypes related to perceived warmth of children thus appeared to diffuse racial/ethnic stereotypes for these three groups, but only to a small degree. For teens, it appeared that negative age-related stereotypes exacerbated endorsement of teens as lazy, which was endorsed at a higher level than for adults across racial/ethnic groups. Black, American Indian/Alaska Native, and Hispanic teens were also considered violence-prone and unintelligent at levels comparable to adult group members.

These patterns support other findings that from age 10, Black children and teens were less likely to be considered innocent and in need of protection, and that their age was over-estimated by on average 4.5 years [45]. Results of this current study suggest that these dehumanizing perceptions of Black older children and teens also extend to their American Indian/Alaska Native and Hispanic same-aged peers. More work is needed using both observational and experimental designs to investigate this further, and to test the most effective ways of countering these dehumanizing stereotypes and their harmful consequences. Perceptions of even small differences between groups can result in differential treatment with deleterious consequences [39, 60]. As well as poorer care and bias from service providers at an interpersonal level across a range of settings, negative stereotypes are also associated with opposition to social policies designed to assist members of that group. The more negative the stereotype, the less likely individuals are to see group members as deserving government assistance or intervention [39, 60, 61]. Thus the observed stereotype levels in this study are likely to influence both individual level service delivery and care received by children and families from stigmatized racial and minority ethnic groups, they are also likely to exert strong influence on policy and procedures at an institutional and community level.

Stereotypes of Asians as more intelligent and hardworking than Whites, and less violence-prone, also persisted in this study towards adults, teens and children. This supports work documenting the ongoing persistence of the ‘model minority’ image of Asians as excessively competent [62] and studious, particularly in science, mathematics and music [63]. The low observed levels of the violence-prone stereotype towards Asians, making them less likely to elicit threat and fear responses from others, could be interpreted as Asians being seen as higher in warmth stereotypes [62]. However, a more likely explanation is that this finding relates to other evidence that shows Asians are seen as deferential and socially weak [63, 64] and lacking in sociability [62, 65].

The results of this current study are of considerable concern given the real world impact of stereotypes on conscious and unconscious behavior [28, 29]. Blacks, American Indian/Alaska Natives and Hispanics were the most stereotyped in this study among White adults, with each of these groups considered low in both warmth and competence dimensions of the stereotype content model (SCM); that is, their stereotypes were evaluatively consistent or univalent [62]. Such univalent stereotypes are associated with the most low-status groups, with those perceived as lacking both warmth and competence most likely to elicit antipathy, anger, contempt, disgust, hate, and resentment [62]. Consistent with wider empirical evidence [30, 31, 33–37] it is thus highly plausible the levels of stereotypes observed in this study contribute to differential treatment and service provider bias for children and families from Black and American Indian/Alaska Native backgrounds and to the racial/ethnic disparities they experience. Negative stereotypes about adult minority group members have enormous implications for child and adolescent health and developmental outcomes as well, given that parents centrally influence opportunities and barriers that minority children will encounter. Unfair treatment towards parents resulting from negative stereotypes may erode parents’ capacity to provide supportive and sensitive care [15], and research indicates that parents’ experiences of discrimination are associated with child health and developmental outcomes [66–68].

The findings from this study of White adults who work or volunteer with children are broadly consistent with nationally representative population data from the GSS that show Black and other minority adults are negatively stereotyped in the U.S. and there has been minimal change in documented levels of stereotyping since 1990 [25, 54, 55]. In this study, notably fewer White respondents (24%) stereotyped Black adults as lazy compared to 32% of White respondents in the 2010 GSS in and 45% in 1990. More respondents in this study (36%) were also likely to consider Blacks as hardworking than in either the 2010 (16%) or 1990 (16%) GSS. Stereotyping of Black adults as violence-prone in this study (52%) was nearly identical to the 1990 GSS (51%), showing both little change over time and little difference in levels of this belief between White adults working and volunteering with children and the wider U.S. population. Strikingly, more respondents in this study (19%) considered Black adults as unintelligent than in the 2010 GSS (13%), although this was fewer than in 1990 (29%). Patterns of change (or lack thereof) in stereotype endorsement towards Hispanics and Asians in this study compared to the 1990 GSS were similar to those for Blacks [61]. In this study far fewer respondents considered either Hispanics (10%) or Asians (3%) lazy than in the 1990 GSS (32% and 15% respectively); “violence-prone” was also endorsed at similar levels in this study and in 1990 for Hispanics (43% v 43%) and far less for Asians (10% v 20%%) in this study compared to 1990. More change was observed in stereotypes of Hispanics as unintelligent in this study, although this was still endorsed by more than one in five respondents (22%) compared to 32% in 1990; only about half as many respondents in this study considered Asians unintelligent (8%) as in 1990 (15%).

A further contribution of this study is data on levels of stereotypes observed for other racial/ethnic groups not included in the GSS, such as Arab Americans and indigenous peoples. American Indians/Alaska Native adults were among the most likely to be considered lazy (31%) and least likely to be considered hardworking (22%), and while considered less violence-prone (29%) than Blacks and Hispanics, this stereotype was endorsed by nearly a third of respondents, and at a similar level as for Arabs. AI/AN adults were also considered unintelligent at similar levels (17%) as both Blacks (19%) and Hispanics (22%) and almost twice as much as Whites. Indigenous peoples are among the most disadvantaged and excluded population groups globally, particularly in post-colonial states such as the U.S. where the legacy of colonization, dispossession and racism continues to impact on indigenous peoples’ lives in substantial ways [69]. Comparable data is limited globally, although Australian data shows Indigenous Australians are considered hardworking by only 20% of Australians compared to 71% for “Australians in general” [70], consistent with the level observed for American Indians/Alaska Natives in this study. Although a small proportion (1.7%) of the overall U.S. population identify as American Indian or Alaska Native, either alone or in combination with one or more other races [71], even less than the 2.7% of the Australian total population who identify as Indigenous, these data reinforce the position of indigenous peoples as among the most excluded, and most invisible population groups. In contrast, however, this study found Pacific Islander/Native Hawaiian adults were among the least likely to be negatively stereotyped, with levels similar to or less than those for Whites. This may reflect the reality that most Americans are less familiar with this population (compared to American Indians and Alaska Natives) due to less personal contact and less salience of this population in U.S. media and culture. A more surprising finding, given global rises in prejudice against Arab and Muslim people in recent years [72, 73], was that in this study Arab Americans were stereotyped at relatively low levels compared to Blacks, Hispanics and American Indians/Alaska Natives. More work is required to investigate stereotype content and prevalence towards both American Indian/Alaska Natives and Arab Americans across a wider sample.

This study had some limitations. Self-report data on racial stereotypes is open to social desirability effects so levels of stereotype endorsement may be under-estimates for stigmatized groups. Yet these self-report methods are widely utilized for measuring explicit racial attitudes and trait endorsement. They also allow for comparison with major surveys such as the GSS. The sampling strategy whereby participants randomly responded to questions for one of the additional race/ethnicity categories (American Indian/Alaska Natives, Asian, Pacific Islanders/Native Hawaiians, Arab) and for one of the non-adult age groups (young children, teens) reduced the available sample for effect measure modification analysis, increasing potential error. Dichotomization of the negative stereotype variable to compare those who responded ‘yes’ with a collapsed ‘neither/counter’ category may lose sensitivity between those endorsing ‘neither’ stereotype and those endorsing the ‘counter’ or positively-valenced stereotype. Sensitivity analysis using 7-point and trichotomous variables for each stereotype were conducted and similar patterns of associations found, with the dichotomous variable used for ease of interpretation and to allow focus on those responding ‘yes’ to the negative stereotype following previous approaches using the GSS [39].

Notwithstanding these limitations, study findings highlight important avenues for policy, practice and research. More work is needed to explore the findings of this study across both observational and experimental studies. Such studies particularly need to consider stereotypes towards American Indian/Alaska Native people, as well as the racial/ethnic groups more commonly included in such investigations. Further disaggregation of the “young children” age group category, for example 0–4 years v 5–8 years following previous studies [45, 46] would also elucidate more specific information regarding the age at which children begin to be racially stereotyped. Extending studies to consider intersections of race/ethnicity, age and gender is also an important area of work, building on strong existing findings regarding intersections across race and gender in relation to stereotype content and stigmatizing social processes and categorization. Replicating use of the unhealthy habits stereotype, as well as further measurement of both warmth and competence stereotype dimensions, and their subsequent emotional and behavioral consequences is also an important area of work. Utilizing measures of implicit bias that reach beyond self-report measures is also important. Such studies are needed both among adults who work and/or volunteer with children as well as among the general population. While this study was not powered sufficiently to enable examination of respondent age, sex, education level and other demographic characteristics as moderators of stereotype endorsement, this also warrants future investigation.

Findings of this study also highlight the critical need for investment in anti-racism interventions targeted towards adults who work and volunteer with children, the families and children with whom they have contact. While documenting inequalities in stereotyping of racial/ethnic groups as in this current study is critically important, it is also essential to generate evidence that can be used to intervene to address these stereotypes, and their impacts, at both a population level to shift overall attitudes and beliefs, and at a local level within community contexts [51, 74]. Repeated documentation of the pervasive and pernicious nature of inequalities, including racism, without identifying modifiable factors and potential solutions holds the danger of reinforcing widely held beliefs in the intractability of injustice [51]. Promising anti-racism and prejudice reduction interventions do currently exist across population, community and individual levels, although far more work is needed to develop a robust evidence base to inform policy and practice in this area. Documenting the effectiveness of such promising interventions on reducing expressions of racism and prejudice amongst majority group members, *and* in improving population health, particularly for children and young people, is a research priority in the U.S and globally. Reducing racism and improving population health requires multi-level action directed at both stigmatized and non-stigmatized groups to enhance coping and resilience of people experiencing racism as well as to change attitudes, behaviors, policies and practices of non-stigmatized people and institutions and systems in the socio-political environment [75, 76]. At a population level, advertising, mass media and educational interventions that attempt to promote positive attitudes and reduce stereotypes towards stigmatized racial/ethnic groups show some promise, such as a recent national anti-discrimination campaign implemented in Australia via a television, digital and out of home advertising campaign [77, 78]. Targeted advertising campaigns have also been shown to improve health outcomes for stigmatized groups, as found in a ‘countermarketing’ campaign that used outdoor advertising to disseminate stark facts about the persistence of racism in the US across two predominantly black New York City (NYC) neighborhoods [79]. At an interpersonal level, interventions exist that improve the ways dominant-group adults interact with racial-minority students [80] and promote positive intergroup contact [81], as well as support groups and other small group interactions to support coping with stigma and develop positive goals for the future [82]. Intrapersonal interventions include counselling, social belonging and values affirmation activities [83] for those from stigmatized racial/ethnic groups, as well as education interventions to raise awareness of both conscious and unconscious bias and stereotyping among non-stigmatized people [84]. As highlighted above, research is needed regarding the effectiveness of these promising interventions in reducing stereotyping and negative racial/ethnic attitudes among adults who work and volunteer with children across a range of settings, and in improving the health of children and families with whom they have contact. With mounting evidence that caregiver and family experiences of racism have detrimental impacts on child health outcomes [67, 68, 85, 86], as well as children’s own experiences of racism [8, 15, 16, 18, 20], this is a critical priority.

This study found high levels of observed stereotypes towards Blacks, American Indian/Alaska Natives and Hispanics with adults from these groups all perceived as being lazy, violence prone, unintelligent, and with unhealthy habits more than Whites by substantial proportions of White adults who work and/or volunteer with children. Conversely, each of these stereotypes was observed at lower levels for Asians adults than for Whites, suggesting persistence of ‘model minority’ images, with low levels of stereotype endorsement also observed for PI/NH adults. Age-related stereotypes diffused racial/ethnic stereotypes to some degree for teens and young children, although negative stereotypes towards Blacks, American Indian/Alaska Natives and Hispanics persisted for young children and adolescents. Findings suggest that White adults who work and/or volunteer with children in the U.S. require ongoing support to counter stereotypes and develop positive racial/ethnic attitudes and beliefs. Such work must reach beyond finding more sophisticated ways of understanding the complexities of prejudice to finding the most effective ways of preventing and addressing prejudice and its consequences for health from childhood through adulthood.

## Supporting information

S1 Fig IRB Exempt Approval Letter. (PDF)

S1 Table Population weighted estimates of mean levels of stereotype endorsement towards adults, by racial group, among White adults who work or volunteer with children. (DOCX)

S2 Table Population weighted estimates of mean levels of stereotype endorsement towards teens, by racial group, among White adults who work or volunteer with children. (DOCX)

S3 Table Population weighted estimates of mean levels of stereotype endorsement towards young children, by racial group, among White adults who work or volunteer with children. (DOCX)

S4 Table Multivariable associations between stereotype endorsement and target racial group among White adults who work or volunteer with children. (DOCX)

S5 Table Population weighted estimates of mean levels of stereotype endorsement towards adults, by racial group, among White adults who work or volunteer with children, by on and off panel. (DOCX)

S1 File Minimal dataset. (DTA)
