# Correction: Stereotyping across intersections of race and age: Racial stereotyping among White adults working with children

**DOI:** 10.1371/journal.pone.0205614

**Published:** 2018-10-05

**Authors:** Naomi Priest, Natalie Slopen, Susan Woolford, Jeny Tony Philip, Dianne Singer, Anna Daly Kauffman, Kathryn Moseley, Matthew Davis, Yusuf Ransome, David Williams

The seventh author's name is spelled incorrectly. The correct name is: Kathryn Moseley.
