# Supplementary material for: Stereotyping across intersections of race and age: Racial stereotyping among White adults working with children
Source: PLoS One. 2018 Sep 12;13(9):e0201696. doi: 10.1371/journal.pone.0201696 (PMC6135395; doi:10.1371/journal.pone.0201696)
Supplement: S1 Table — (DOCX) [file pone.0201696.s002.docx]

Supplemental Table 1 Population weighted estimates of mean levels of stereotype endorsement towards adults, by racial group, among White adults who work or volunteer with children*

|  | **White**  **M (95% CI)** | **Afr. Am.**  **M (95% CI)** | **Hispanic**  **M (95% CI)** | **AI/AN**  **M (95% CI)** | **Asian Am.**  **M (95% CI)** | **PI/NH**  **M (95% CI)** | **Arab Am.**  **M (95% CI)** |
| --- | --- | --- | --- | --- | --- | --- | --- |
|  | **n=1004** | **n=1005** | **n=1004** | **n=244** | **n=249** | **n=265** | **n=241** |
| Hardworking or Lazy | 3.06 (2.93, 3.19) | 3.74 (3.58, 3.9) | 3.11 (2.96, 3.26) | 4.1 (3.83, 4.37) | 2.71 (2.39, 3.02) | 3.33 (3.1, 3.55) | 3.56 (3.21, 3.92) |
| Not violence prone or violence prone | 3.93 (3.82, 4.04) | 4.6 (4.47, 4.74) | 4.4 (4.27, 4.53) | 4.13 (3.89, 4.37) | 3.3 (3.02, 3.58) | 3.65 (3.46, 3.84) | 3.98 (3.71, 4.25) |
| Intelligent or Unintelligent | 3.43 (3.31, 3.55) | 3.82 (3.71, 3.93) | 3.93 (3.81, 4.04) | 3.88 (3.69, 4.08) | 3.22 (2.93, 3.5) | 3.58 (3.35, 3.82) | 3.67 (3.41, 3.92) |
| Healthy or Unhealthy habits | 3.85 (3.72, 3.97) | 4.28 (4.16, 4.4) | 4.19 (4.08, 4.31) | 4.34 (4.11, 4.58) | 3.4 (3.08, 3.71) | 3.83 (3.67, 3.98) | 3.69 (3.45, 3.92) |

*Range 1-7, higher score=more negative stereotype
