# Supplementary material for: Stereotyping across intersections of race and age: Racial stereotyping among White adults working with children
Source: PLoS One. 2018 Sep 12;13(9):e0201696. doi: 10.1371/journal.pone.0201696 (PMC6135395; doi:10.1371/journal.pone.0201696)
Supplement: S2 Table — (DOCX) [file pone.0201696.s003.docx]

Supplemental Table 2 Population weighted estimates of mean levels of stereotype endorsement towards teens, by racial group, among White adults who work or volunteer with children*

|  | **White**  **M (95% CI)** | **Afr. Am.**  **M (95% CI)** | **Hispanic**  **M (95% CI)** | **AI/AN**  **M (95% CI)** | **Asian Am.**  **M (95% CI)** | **PI/NH**  **M (95% CI)** | **Arab Am.**  **M (95% CI)** |
| --- | --- | --- | --- | --- | --- | --- | --- |
|  | **n=491** | **n=494** | **n=493** | **n=123** | **n=127** | **n=129** | **n=133** |
| Hardworking or Lazy | 3.98 (3.76, 4.19) | 4.32 (4.1, 4.53) | 3.97 (3.77, 4.17) | 4.46 (4.05, 4.87) | 3.37 (3, 3.74) | 3.76 (3.46, 4.06) | 3.95 (3.56, 4.34) |
| Not violence prone or violence prone | 4 (3.84, 4.15) | 4.55 (4.37, 4.74) | 4.44 (4.27, 4.61) | 4.12 (3.8, 4.44) | 3.6 (3.2, 4) | 3.76 (3.49, 4.04) | 4.11 (3.81, 4.42) |
| Intelligent or Unintelligent | 3.42 (3.27, 3.57) | 3.78 (3.6, 3.95) | 3.81 (3.63, 3.99) | 3.61 (3.32, 3.9) | 3.27 (2.78, 3.76) | 3.58 (3.27, 3.88) | 3.64 (3.25, 4.03) |
| Healthy or Unhealthy habits | 3.91 (3.71, 4.12) | 4.15 (3.96, 4.34) | 4.13 (3.94, 4.31) | 4.03 (3.62, 4.43) | 3.67 (3.15, 4.19) | 3.86 (3.57, 4.15) | 3.74 (3.35, 4.12) |

*Range 1-7, higher score=more negative stereotype
