# Supplementary material for: Stereotyping across intersections of race and age: Racial stereotyping among White adults working with children
Source: PLoS One. 2018 Sep 12;13(9):e0201696. doi: 10.1371/journal.pone.0201696 (PMC6135395; doi:10.1371/journal.pone.0201696)
Supplement: S3 Table — (DOCX) [file pone.0201696.s004.docx]

Supplemental Table 3 Population weighted estimates of mean levels of stereotype endorsement towards young children, by racial group, among White adults who work or volunteer with children*

|  | **White**  **M (95% CI)** | **Afr. Am.**  **M (95% CI)** | **Hispanic**  **M (95% CI)** | **AI/AN**  **M (95% CI)** | **Asian Am.**  **M (95% CI)** | **PI/NH**  **M (95% CI)** | **Arab Am.**  **M (95% CI)** |
| --- | --- | --- | --- | --- | --- | --- | --- |
|  | **n=491** | **n=494** | **n=493** | **n=123** | **n=127** | **n=129** | **n=133** |
| Hardworking or Lazy | 3.75 (3.56, 3.94) | 3.96 (3.78, 4.14) | 3.85 (3.68, 4.01) | 4.02 (3.73, 4.31) | 3.62 (3.09, 4.16) | 3.75 (3.53, 3.97) | 3.79 (3.59, 3.99) |
| Not violence prone or violence prone | 3.69 (3.53, 3.85) | 4.1 (3.91, 4.29) | 3.96 (3.78, 4.14) | 3.94 (3.55, 4.33) | 3.3 (2.91, 3.69) | 3.74 (3.47, 4.01) | 3.92 (3.65, 4.18) |
| Intelligent or Unintelligent | 3.56 (3.42, 3.7) | 3.85 (3.73, 3.97) | 3.85 (3.74, 3.97) | 3.9 (3.64, 4.16) | 3.34 (3.03, 3.65) | 3.62 (3.4, 3.84) | 3.69 (3.45, 3.94) |
| Healthy or Unhealthy habits | 4.03 (3.84, 4.22) | 4.36 (4.2, 4.52) | 4.3 (4.16, 4.44) | 4.53 (4.2, 4.86) | 3.57 (3.21, 3.93) | 4.05 (3.74, 4.35) | 4.07 (3.68, 4.46) |

*Range 1-7, higher score=more negative stereotype
