# Supplementary material for: Stereotyping across intersections of race and age: Racial stereotyping among White adults working with children
Source: PLoS One. 2018 Sep 12;13(9):e0201696. doi: 10.1371/journal.pone.0201696 (PMC6135395; doi:10.1371/journal.pone.0201696)
Supplement: S4 Table — (DOCX) [file pone.0201696.s005.docx]

Supplemental Table 4 Multivariable associations between stereotype endorsement and target racial group among White adults who work or volunteer with children*

|  | **Hardworking/Lazy**  **Adjusted b (95% CI)** | **Not violence prone/Violence prone**  **Adjusted b (95% CI)** | **Intelligent/Unintelligent**  **Adjusted b (95% CI)** | **Unhealthy habits/Healthy habits**  **Adjusted b (95% CI)** |
| --- | --- | --- | --- | --- |
| Racial group |  |  |  |  |
| Afr. Am | 0.68 (0.53, 0.83) *** | 0.67 (0.54, 0.81) *** | 0.39 (0.28, 0.49) *** | 0.43 (0.3, 0.57) *** |
| Hispanic | 0.05 (-0.1, 0.19) | 0.47 (0.35, 0.59) *** | 0.49 (0.37, 0.61) *** | 0.35 (0.2, 0.5) *** |
| AI/AN | 0.98 (0.72, 1.24) *** | 0.09 (-0.11, 0.29) | 0.43 (0.24, 0.63) *** | 0.46 (0.22, 0.7) *** |
| Asian Am | -0.37 (-0.68, -0.07) * | -0.61 (-0.93, -0.3) *** | -0.19 (-0.45, 0.06) | -0.47 (-0.8, -0.14) ** |
| PI/NH | 0.32 (0.15, 0.5) *** | -0.22 (-0.4, -0.04) * | 0.16 (0.01, 0.3) * | 0.01 (-0.16, 0.18) |
| Arab Am | 0.52 (0.18, 0.86) *** | 0.07 (-0.13, 0.28) | 0.24 (0.08, 0.4) ** | -0.14 (-0.34, 0.06) |
| Racial group X Age group |  |  |  |  |
| Child X Afr. Am | -0.47 (-0.64, -0.3) *** | -0.27 (-0.45, -0.08) ** | -0.1 (-0.23, 0.03) | -0.1 (-0.28, 0.07) |
| Child X Hispanic | 0.05 (-0.11, 0.2) | -0.2 (-0.37, -0.03) * | -0.2 (-0.33, -0.07) ** | -0.07 (-0.25, 0.11) |
| Child X AI/AN | -0.75 (-1.07, -0.43) *** | 0.03 (-0.24, 0.3) | -0.09 (-0.27, 0.1) | 0.03 (-0.24, 0.3) |
| Child X Asian Am | 0.26 (-0.24, 0.77) * | 0.27 (-0.06, 0.59) * | -0.01 (-0.28, 0.26) | -0.01 (-0.39, 0.37) |
| Child X PI/NH | -0.31 (-0.58, -0.04) * | 0.34 (0.05, 0.63) | -0.12 (-0.28, 0.05) | -0.01 (-0.24, 0.21) |
| Teen X Arab Am | -0.45 (-0.87, -0.03) | 0.2 (-0.06, 0.45) | -0.04 (-0.26, 0.17) | 0.27 (-0.01, 0.56) |
| Teen X Afr. Am | -0.35 (-0.53, -0.18) | -0.11 (-0.29, 0.08) | -0.01 (-0.15, 0.12) * | -0.19 (-0.36, -0.02) ** |
| Teen X Hispanic | -0.04 (-0.24, 0.15) | -0.02 (-0.16, 0.13) | -0.1 (-0.24, 0.04) | -0.13 (-0.31, 0.04) |
| Teen X AI/AN | -0.58 (-0.94, -0.23) *** | 0 (-0.3, 0.3) | -0.25 (-0.53, 0.03) | -0.39 (-0.82, 0.03) |
| Teen X Asian Am | -0.27 (-0.72, 0.17) | 0.13 (-0.32, 0.58) | 0.03 (-0.31, 0.38) | 0.23 (-0.19, 0.65) |
| Teen X PI/NH | -0.45 (-0.73, -0.18) *** | 0.08 (-0.16, 0.31) | 0.06 (-0.14, 0.26) | -0.01 (-0.27, 0.25) |
| Teen X Arab Am | -0.55 (-0.97, -0.14) ** | 0.09 (-0.24, 0.41) | -0.05 (-0.34, 0.24) | -0.03 (-0.3, 0.24) |
| Cons | 3.23 (2.65, 3.8) | 4.09 (3.65, 4.54) | 3.71 (3.26, 4.16) | 3.86 (3.47, 4.24) |

*Range 1-7, higher score=more negative stereotype

All models adjusted for respondent age, sex, education

*p≤0.05, **p≤0.01, ***p≤0.001
