# Supplementary material for: Stereotyping across intersections of race and age: Racial stereotyping among White adults working with children
Source: PLoS One. 2018 Sep 12;13(9):e0201696. doi: 10.1371/journal.pone.0201696 (PMC6135395; doi:10.1371/journal.pone.0201696)
Supplement: S5 Table — (DOCX) [file pone.0201696.s006.docx]

Supplemental Table 5 Population weighted estimates of mean levels of stereotype endorsement towards adults, by racial group, among White adults who work or volunteer with children, by on and off panel*

|  | **White** | **Afr. Am.** | **Hispanic** | **AI/AN** | **Asian Am.** | **PI/NH** | **Arab Am.** |
| --- | --- | --- | --- | --- | --- | --- | --- |
| **Hardworking or Lazy** | **M (95% CI)** | **M (95% CI)** | **M (95% CI)** | **M (95% CI)** | **M (95% CI)** | **M (95% CI)** | **M (95% CI)** |
| On panel | 3.06 (2.92, 3.19) | 3.64 (3.5, 3.78) | 3.11 (2.95, 3.26) | 3.9 (3.65, 4.14) | 2.7 (2.38, 3.03) | 3.32 (3.1, 3.55) | 3.31 (3.07, 3.56) |
| Off panel | 3.18 (3.06, 3.31) | 3.74 (3.58, 3.9) | 3.11 (2.97, 3.25) | 4.1 (3.83, 4.38) | 2.8 (2.5, 3.09) | 3.36 (3.08, 3.63) | 3.57 (3.2, 3.93) |
